# Self-Supported Cu/Fe_3_O_4_ Hierarchical Nanosheets on Ni Foam for High-Efficiency Non-Enzymatic Glucose Sensing

**DOI:** 10.3390/nano15040281

**Published:** 2025-02-12

**Authors:** Jing Xu, Hairui Cai, Ke Yu, Jie Hou, Zhuo Li, Xiaoxiao Zeng, Huijie He, Xiaojing Zhang, Di Su, Shengchun Yang

**Affiliations:** 1 MOE Key Laboratory for Non-Equilibrium Synthesis and Modulation of Condensed Matter, Key Laboratory of Shaanxi for Advanced Materials and Mesoscopic Physics, State Key Laboratory for Mechanical Behavior of Materials, School of Physics, Xi’an Jiaotong University, No. 28 West Xianning Road, Xi’an 710049, China; xujing1@xpu.edu.cn (J.X.); sherlock_hou@stu.xjtu.edu.cn (J.H.); zxxkeep@stu.xjtu.edu.cn (X.Z.); a592003062@stu.xjtu.edu.cn (H.H.); jingfang114love@xjtu.edu.cn (X.Z.); ysch1209@xjtu.edu.cn (S.Y.); 2 School of Mechanical and Electrical Engineering, Xi’an Polytechnic University, No. 19 Jinhua South Road, Xi’an 710048, China; y19145514@xauat.edu.cn; 3 School of Electrical Engineering, Xi’an Jiaotong University, Xi’an 710049, China; lizhuo3007@stu.xjtu.edu.cn; 4 Shaanxi Hydrogen Energy Research Institute Co., Ltd., Xi’an 712046, China; sudi600@sina.com

**Keywords:** Cu, Fe_3_O_4_, hierarchical nanosheets, non-enzymatic glucose sensor

## Abstract

Electrochemical glucose sensors are vital for clinical diagnostics and the food industry, where accurate detection is essential. However, the limitations of glucose oxidase (GOx)-based sensors, such as complex preparation, high cost, and environmental sensitivity, highlight the need for non-enzymatic sensors that directly oxidize glucose at the electrode surface. In this study, a self-supporting hierarchical Cu/Fe_3_O_4_ nanosheet electrode was successfully fabricated by in situ growth on Ni Foam using a hydrothermal method, followed by annealing treatment. The Cu/Fe_3_O_4_ hierarchical nanosheet structure, with its large surface area, provides abundant active sites for electrocatalysis, while the strong interactions between Cu/Fe_3_O_4_ and Ni Foam enhance electron transfer efficiency. This novel electrode structure demonstrates exceptional electrochemical performance for non-enzymatic glucose sensing, with an ultrahigh sensitivity of 12.85 μA·μM^−1^·cm^−2^, a low detection limit of 0.71 μM, and a linear range extending up to 1 mM. Moreover, the Cu/Fe_3_O_4_/NF electrode exhibits excellent stability, a rapid response (~3 s), and good selectivity against interfering substances such as uric acid, ascorbic acid, H_2_O_2_, urea, and KCl. It also shows strong reliability in analyzing human serum samples. Therefore, Cu/Fe_3_O_4_/NF holds great promise as a non-enzymatic glucose sensor, and this work offers a valuable strategy for the design of advanced electrochemical electrodes.

## 1. Introduction

Electrochemical glucose sensors have garnered increasing attention recently due to their critical role in providing accurate and timely glucose detection toward clinical diagnostics and the food industry [1,2,3]. The high sensitivity and selectivity of glucose oxidase made it the preferred method of glucose detection over the past decades [4,5,6]. Traditional enzymatic glucose sensors rely on enzymes, such as glucose oxidase (GOx), to catalyze glucose oxidation. Although enzymatic glucose sensors provide high sensitivity and specificity, their practical application is still limited because of the complex preparation processes, high costs, and susceptibility to deactivation caused by environmental factors such as temperature and pH [7,8]. Thus, a crucial strategy is developing a non-enzymatic glucose sensor with an electrochemical process that directly oxidates glucose at the electrode surface. The key to developing effective non-enzymatic glucose sensors depends on selecting materials with high catalytic activity and stability [9,10,11,12].

In recent years, non-noble-metal copper (Cu) and its derivatives have been widely studied. They benefit from their good glucose sensing properties, wide linear detection range, electrical conductivity, and stability. Nanoparticles, nanowires, nanorods, and other nanostructures made of Cu-based electrode materials are frequently used to improve glucose sensing performance by increasing specific surface area [13,14,15,16,17]. Yuan et al. in situ-synthesized a three-dimensional (3D) binder-free CuO nanosheet-encapsulated nanofilm on a Cu substrate, exhibiting an excellent non-enzymatic glucose oxidation with a sensitivity of 4201 μA·cm^−2^·mM^−1^ and detection limit of 0.5 μM as well as response time of 0.7 s [18]. Lin et al. prepared novel Cu/Cu_2_O/CuO ternary composite hollow spheres using an aerosol furnace reactor, revealing a good sensitivity of 8726 μA·cm^−2^·mM^−1^ and anti-interference performance due to the synergistic effect of the ternary composite with multiple Cu redox coupling interfaces and large specific surface area [19]. Wei et al. successfully synthesized Cu_2_O/CuO nanowire arrays modified by Cu nanosheets on Cu foil using in situ growth and a continuous ion layer adsorption reaction, which exhibited a high sensitivity of 4262 μA·cm^−2^·mM^−1^ with a linear detection range extending from 0.002 mM to 4.096 mM and superb selectivity, reproducibility, and stability benefit from the increased active specific surface area of the electrode with Cu nanosheet modification [20]. However, all previously mentioned Cu-based materials have inherent disadvantages, such as insufficient stability, poor repeatability, susceptibility to chloride ion poisoning, high operating conditions, and safety risks. It has been reported that combining different materials with complementary properties can generate synergistic effects, enhancing catalytic activity, improving stability, reducing overpotentials, increasing selectivity, and enabling tunable properties of non-enzymatic glucose sensors.

Due to low toxicity, biocompatibility, superparamagnetism, and catalytic activity, Fe_3_O_4_ has received considerable interest. Fe_3_O_4_ has a high level of chemical activity and simultaneously contains Fe^2+^ and Fe^3+^, in which Fe^3+^ possesses a higher propensity to oxidize glucose to produce electrons catalytically [21]. Meanwhile, since Fe_3_O_4_ can form reactive oxygen species that can further promote glucose oxidation, it is a more appealing functional material for non-enzymatic glucose detection [22,23]. For example, Zhang synthesized Fe_3_O_4_ nanosphere materials for non-enzymatic glucose sensors, demonstrating a range of impressive electrochemical properties, including a high sensitivity of 6560 μA mM^−1^ cm^−2^ (linear detection range of glucose concentrations from 0.1 to 1.1 mM), detection limit of 33 μM (S/N = 3), and good long-term stability [23]. It can be seen that combining Fe_3_O_4_ with Cu leverages the high electrical conductivity of Cu while preserving the catalytic activity of Fe_3_O_4_. This synergy results in lower overpotentials and higher current responses, making it an ideal approach for practical glucose detection.

In this work, we successfully synthesized Cu/Fe_3_O_4_ layered double hydroxide (CuFe-LDH) nanosheets in situ on a nickel foam substrate using a hydrothermal method. After annealing, the hierarchical Cu/Fe_3_O_4_ composite nanosheets on a nickel foam substrate (Cu/Fe_3_O_4_/NF) electrode material were obtained. Several characterization techniques were used to characterize the Cu/Fe_3_O_4_ composite nanosheet properties. The resulting Cu/Fe_3_O_4_ nanosheets possess a mesoporous structure, facilitating extensive contact with glucose. Thanks to the synergistic effects between the Cu and Fe_3_O_4_ components, the Cu/Fe_3_O_4_/NF electrode exhibited an ultrahigh sensitivity of 12.85 μA·μM^−1^·cm^−2^ and a low detection limit of 0.71 μM.

## 2. Experimental Section

### 2.1. Chemicals and Reagents

Cu(NO_3_)_2_·3H_2_O, Fe(NO_3_)_3_·9H_2_O, sodium hydroxide (NaOH), ethylene glycol (EG, (CH_2_OH)_2_), ethyl alcohol (C_2_H_5_OH), ascorbic acids (AAs, C_6_H_8_O_6_), uric acids (UAs, C_5_H_4_N_4_O_3_), potassium chloride (KCl), hydrogen peroxide (H_2_O_2_), urea (CH_4_N_2_O), and glucose were purchased from Sinopharm Chemical Co., Ltd., Beijing, China. Nickel foam (NF) was obtained from Suzhou Shengernuo Technology Co., Ltd., Su Zhou, China. Deionized water (18.2 MΩ·cm) was purified using the Ulupure system in all preparations. The experiment used chemical reagents that were all analytically pure and without practicing further purification.

### 2.2. Synthesis of CuFe-LDH/NF Precursor

Quantitative Cu(NO_3_)_2_·3H_2_O, Fe(NO_3_)_3_·9H_2_O, and 61 mM of urea were added to 15 mL of the (CH_2_OH)_2_ solution and uniformly dispersed by ultrasonic treatment for 30 min. Meanwhile, a nickel foam piece with the size of 2 × 3 cm was acid-washed to remove impurities and transferred to the above solution. Then, the mixture was migrated to the stainless steel autoclave and heated to 180 °C for 24 h. After the reaction finished, the product was alternately washed with ethyl alcohol and deionized water, and then vacuum-dried at 60 °C for 24 h, obtaining the target CuFe-LDH/NF. The electrode materials obtained by adding Cu(NO_3_)_2_·3H_2_O and Fe(NO_3_)_3_·9H_2_O in molar ratios of 1:1, 1:2, and 2:1 are Cu1Fe1-LDH/NF, Cu1Fe2-LDH/NF, and Cu2Fe1-LDH/NF, respectively.

### 2.3. Synthesis of Cu/Fe_3_O_4_/NF

The CuFe-LDH/NF precursors were heated at 350 °C in a H_2_/Ar atmosphere for 2 h, forming hierarchical Cu/Fe_3_O_4_ composite nanosheets on a nickel foam substrate. Cu/Fe_3_O_4_/NF(1:1), Cu/Fe_3_O_4_/NF(1:2), and Cu/Fe_3_O_4_/NF(2:1) were derived from Cu1Fe1-LDH/NF, Cu1Fe2-LDH/NF, and Cu2Fe1-LDH/NF, respectively.

### 2.4. Material Characterization

A Bruker D8 ADVANCE X-ray diffractometer (XRD) equipped with Cu Kα radiation (λ = 1.54 Å) was devoted to characterizing the crystal structure and phase composition of the samples. The chemical states of the sample were characterized by X-ray photoelectron spectroscopy (XPS, Thermo Fisher ESCALAB Xi+, Waltham, MA, USA) using an Al Kα X-ray source with a power of 400 W. The vacuum system performance was 5 × 10^−10^ mbar, and the standard C 1s peak was set at 284.8 eV. The total acquisition time was 3 minutes 45.4 seconds with 10 scans, a spot size of 500 μm, and standard lens mode. The analyser mode was CAE with a pass energy of 20.0 eV, an energy step size of 0.100 eV, and a total of 451 energy steps. Field-emission scanning electron microscopy (FESEM) using a JEOL JSM-7000F instrument (Tokyo, Japan) operating at an acceleration voltage of 15 kV was used to observe the morphology of the products. Transmission electron microscopy (TEM) and energy-dispersive X-ray spectroscopy (EDS) using a JEOL JEM-F200 device (Tokyo, Japan) were utilized to investigate the microstructure of the products.

### 2.5. Electrochemical Measurements

The as-prepared samples were directly chosen as the working electrode without additional processing. A common three-electrode test system (CHI-660C, Shanghai Chenhua, China) was applied to conduct room-temperature electrochemical measurements, in which the 0.1 M NaOH solution served as the electrolyte, an Ag/AgCl electrode served as the reference electrode, a platinum net served as the counter electrode, and the Cu/Fe_3_O_4_/NF working electrode was fixed using a platinum plate electrode clip. The magnetic stirring treatment was essential when measuring the glucose concentration change based on chronoamperometry and cyclic voltammetry (CV), as it ensured the accuracy of the glucose concentration value close to the electrode. CV tests were conducted in a 0.1 M NaOH solution with glucose concentrations ranging from 0 to 1001 μM, at a scan rate of 50 mV/s and a potential range of 0 to 0.8 V vs. Ag/AgCl. Chronoamperometry tests were carried out at 0.55 V, 0.6 V, and 0.65 V. The electrochemical impedance spectrum (EIS) of NF and Cu/Fe_3_O_4_/NF(1:1) was obtained at 0.6 V vs. Ag/AgCl from 10^−1^ to 10^5^ Hz with an AC amplitude of 5 mV. In the test of real human blood serum, different volumes of human serum (with a blood glucose concentration of 4.07 mM, as measured by a biochemical analyzer) were added to 20 mL of 0.1 M KOH electrolyte solution, and we subsequently evaluated CV curves and chronoamperometry.

## 3. Results and Discussion

### 3.1. Characterizations of Structures and Morphologies

**Figure 1:**
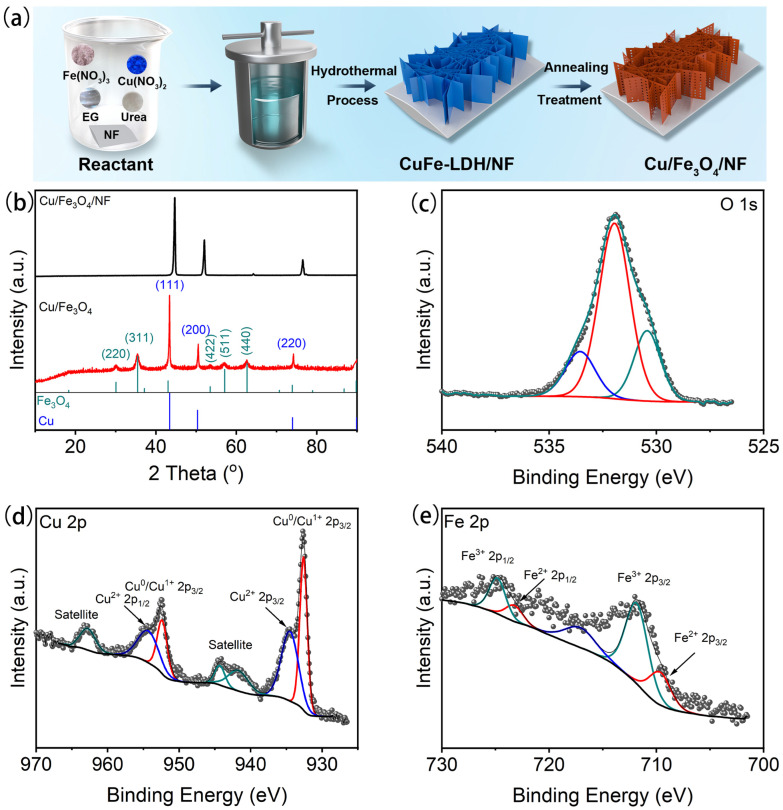
(**a**) The schematic diagram of the Cu/Fe_3_O_4_/NF sample synthesis; the XRD patterns (**b**) and O 1s (**c**), Cu 2p (**d**), and Fe 2p (**e**) XPS spectra of the as-prepared Cu/Fe_3_O_4_/NF nanosheets.

The synthesis of Cu/Fe_3_O_4_/NF follows the process depicted in Figure 1a, with the target sample obtained through hydrothermal synthesis and annealing treatment. XRD methods were utilized to characterize the crystal structure and phase composition of the as-prepared samples. As shown in Figure 1b, the Cu/Fe_3_O_4_/NF sample exhibits only the characteristic peak of Ni, which is attributed to the dominant XRD signal from the nickel foam substrate, overshadowing the peaks from the surface materials. To address this, we further delaminated the sample from the nickel foam substrate and performed XRD analysis on the delaminated material. As illustrated in Figure 1b, the dominant diffraction peaks at 43.4°, 50.5°, and 74.2° corresponding to (111), (200), and (220) lattice planes were detected following the standard cubic structure of Cu crystal (JCPDS no. 01-1241). The Fe_3_O_4_ crystal (JCPDS no. 01-1111) was identified by the remaining diffraction peaks at 30.3°, 35.5°, 52.2°, 53.6°, and 62.5°, corresponding to the (220), (311), (422), (511), and (440) lattice planes, respectively. Also, the obtained products were only Cu/Fe_3_O_4_ composite nanosheets, as evidenced by the absence of any peaks representing other impurities.

XPS analysis was used to investigate further the composition and valence of the as-prepared Cu/Fe_3_O_4_/NF. As seen in Figure 1c, the characteristic peaks at 530.4 eV, 531.5 eV, and 533.1 eV were attributed to the lattice oxygen in Fe_3_O_4_ crystal (530.4 eV) and the oxygen adsorbed on the surface of Fe_3_O_4_ (531.5 eV, 533.1 eV), respectively. As depicted in Figure 1d, the distinctive peaks at 933.5 eV and 953.4 eV were assigned to core-level Cu 2p_3/2_ and Cu 2p_1/2_ of CuO, suggesting the existence of CuO [24,25]. Meanwhile, two satellite peaks that emerged at 963 eV and 943.8 eV concurrently validated the CuO state. It is common knowledge that the oxidation state of copper is typically visible in XPS analysis because copper oxidizes readily in the air, resulting in unavoidable oxidation toward the surface of an as-synthesized catalyst. Given that the Cu 2p signal alone does not allow for a clear distinction between Cu^0^ and Cu^1+^, an analysis of the Cu LMM spectrum was conducted. As shown in Figure S1 and Table S1, it confirmed the coexistence of Cu^0^, Cu^1+^, and Cu^2+^ species on the surface of the synthesized material. Additionally, the Fe 2p XPS spectrum in Figure 1e shows peaks at 713.2 eV/726.8 eV and 709.8 eV/723.6 eV, which were associated with Fe(III) and Fe(II), further confirming the presence of Fe_3_O_4_ in the composition [24,26,27]. Since XPS analyzes information from the sample surface within a depth of ~10 nm, combined with the results from XRD and XPS, it can be concluded that the as-prepared electrode consists of Cu and Fe_3_O_4_.

**Figure 2:**
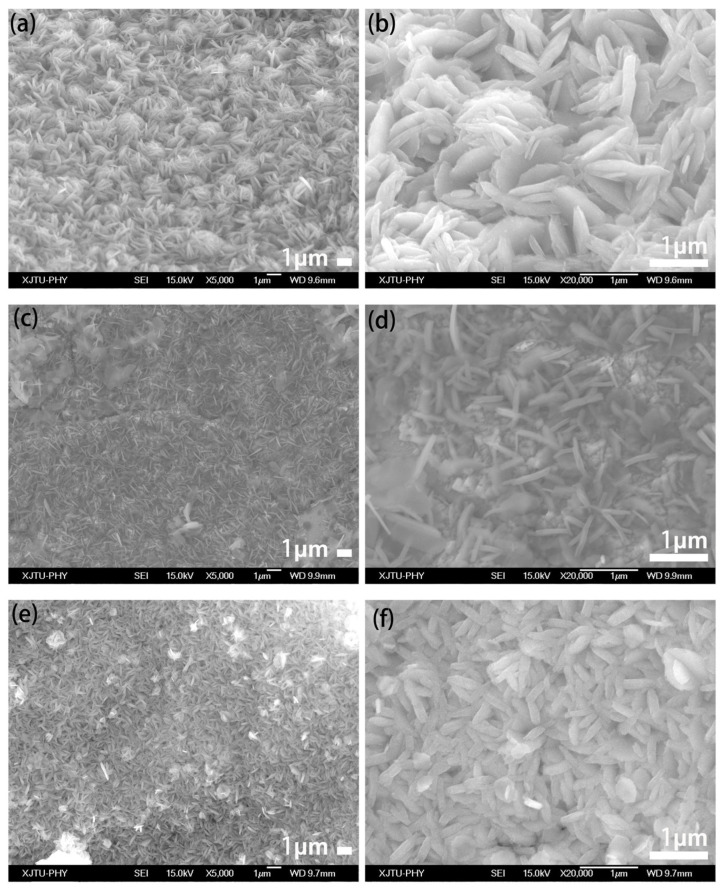
SEM images of the as-prepared CuFe-LDH/NF: (**a**,**b**) Cu1Fe1-LDH/NF; (**c**,**d**) Cu2Fe1-LDH/NF; (**e**,**f**) Cu1Fe2-LDH/NF.

**Figure 3:**
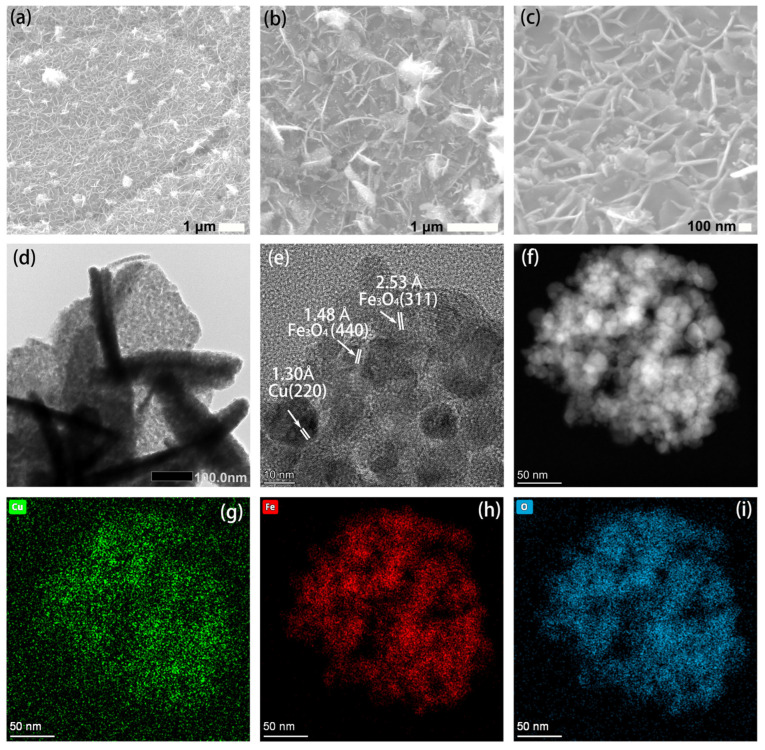
SEM images of the Cu/Fe_3_O_4_/NF (**a**–**c**); a TEM image of the Cu/Fe_3_O_4_/NF (**d**); an HRTEM image of the Cu/Fe_3_O_4_/NF (**e**); an HAADF-STEM image of Cu/Fe_3_O_4_/NF (**f**); and the corresponding elemental mappings of Cu (**g**), Fe (**h**), and O (**i**).

Figure 2 presents the SEM images of the prepared CuFe-LDH/NF precursors. It can be observed that the three CuFe-LDH/NF precursors (Cu1Fe1-LDH/NF, Cu2Fe1-LDH/NF, and Cu1Fe2-LDH/NF) have similar microstructures, with nanosheets grown on the surface of the nickel foam substrate. Among these, Cu1Fe1-LDH/NF displays dense and uniform nanosheets with a thickness of approximately 100 nm, whereas Cu2Fe1-LDH/NF and Cu1Fe2-LDH/NF feature more sparse and thinner nanosheet structures. This variation in morphology may be attributed to changes in the metal molar ratio, which alter the deposition rate and growth mechanism of the two metals during hydroxide formation, ultimately influencing the morphology. After annealing, the Cu/Fe_3_O_4_/NF retains a nanosheet morphology similar to that of the precursor CuFe-LDH/NF, with a thickness of approximately 30 nm, as shown in Figure 3a–c. Additionally, the TEM image in Figure 3d reveals a distinct mesoporous structure on the surface of the Cu/Fe_3_O_4_ nanosheets, which is advantageous for providing sufficient active sites for glucose sensor detection. The HRTEM image in Figure 3e further confirms the presence of Cu and Fe_3_O_4_, consistent with the XRD and XPS results. Furthermore, the HAADF image clearly shows that the nanosheets are composed of nanoparticles with a ~10 nm size, indicating that the fabricated Cu/Fe_3_O_4_ nanosheets possess a hierarchical structure. The elemental distribution within the Cu/Fe_3_O_4_ nanosheets was analyzed using energy-dispersive spectroscopy (EDS), as shown in Figure 3f–i, where Cu, Fe, and O are uniformly distributed throughout the Cu/Fe_3_O_4_ nanostructures. And the loading amount of Cu/Fe_3_O_4_ was approximately 1.5 mg cm^−2^ (measured by ICP-MS test, Table S2) in Cu/Fe_3_O_4_/NF(1:1). Overall, we successfully prepared Cu/Fe_3_O_4_ composite nanosheets with a hierarchical and mesoporous structure.

### 3.2. Electrochemical Performance

**Figure 4:**
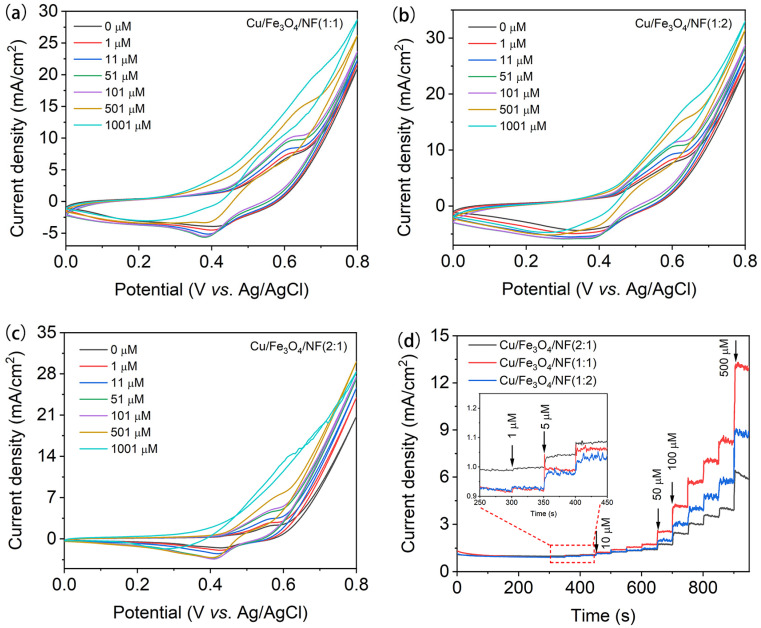
CV curves of the Cu/Fe_3_O_4_/NF(1:1) (**a**), Cu/Fe_3_O_4_/NF(1:2) (**b**), and Cu/Fe_3_O_4_/NF(2:1) (**c**) electrodes in 0.1 M NaOH solution with the concentrations of glucose from 0 to 1001 μM; (**d**) amperometric response of the Cu/Fe_3_O_4_/NF electrode with the successive addition of glucose at 0.6 V (vs. Ag/AgCl).

The electrocatalytic activity of Cu/Fe_3_O_4_/NF for glucose oxidation was assessed using cyclic voltammetry (CV) as a non-enzymatic glucose sensor. Figure 4a–c display the CV curves of Cu/Fe_3_O_4_/NF(1:1), Cu/Fe_3_O_4_/NF(2:1), and Cu/Fe_3_O_4_/NF(1:2) in 0.1 M NaOH solution at various glucose concentrations. It is clear that the glucose oxidation peak for all three samples appears around 0.6 V, and this peak shifts to the right as the glucose concentration increases. Figure 4d displays the amperometric response curve of the as-prepared samples at 0.6 V (vs. Ag/AgCl) following the continuous changes in the glucose concentrations. It reveals that Cu/Fe_3_O_4_/NF(1:1) and Cu/Fe_3_O_4_/NF(1:2) show highly sensitive current responses at very low glucose concentrations (1 μM), whereas Cu/Fe_3_O_4_/NF(2:1) does not exhibit a noticeable current response. As the glucose concentration increases, the current signals for all three samples progressively enhance, with Cu/Fe_3_O_4_/NF(1:1) exhibiting the strongest current response and a more distinct linear increase. Therefore, Cu/Fe_3_O_4_/NF(1:1) was selected for the subsequent glucose performance tests.

**Figure 5:**
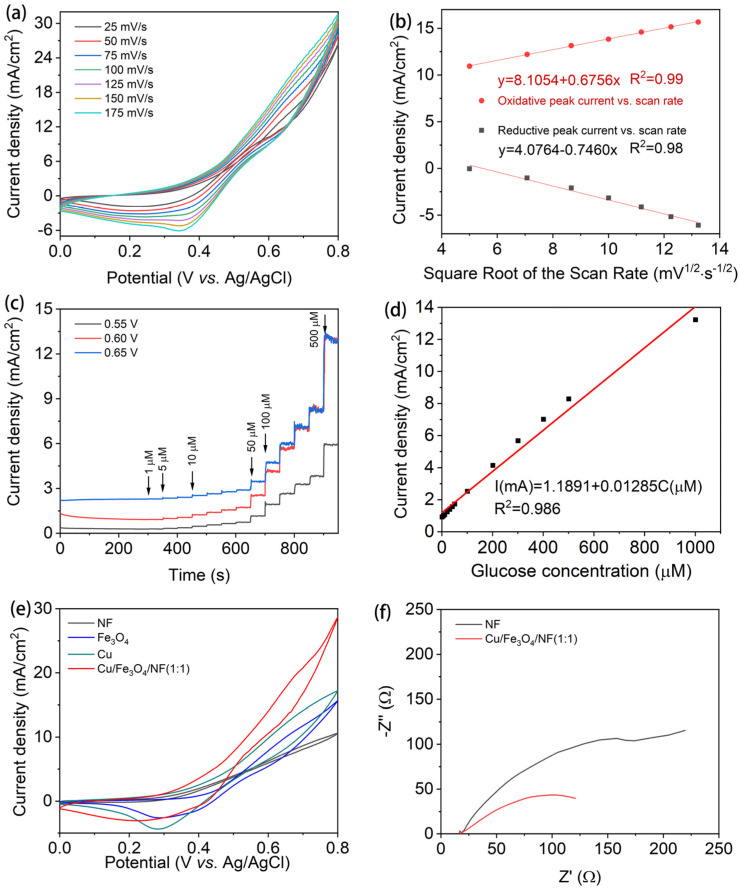
(**a**) CV curves of the Cu/Fe_3_O_4_/NF(1:1) electrode at scan rates of 5~175 mV/s in 0.1 M NaOH solution; (**b**) the plot of the oxidative and reductive peak current vs. scan rate; (**c**) amperometric response of the Cu/Fe_3_O_4_/NF(1:1) electrode at multiple potentials (+0.55 V, +0.60 V, +0.65 V) vs. Ag/AgCl with the successive addition of glucose; (**d**) the corresponding calibration curve of current vs. glucose concentration at the Cu/Fe_3_O_4_/NF(1:1) electrode; (**e**) CV curves of NF, Fe_3_O_4_, Cu, and Cu/Fe_3_O_4_/NF(1:1); (**f**) electrochemical impedance profiles of NF and Cu/Fe_3_O_4_/NF(1:1).

The CV curves of the Cu/Fe_3_O_4_/NF(1:1) electrode at different scan rates of 5~175 mV/s in 0.1 M NaOH solution are illustrated in Figure 5a. The current intensity of the Cu/Fe_3_O_4_/NF(1:1) electrode increased with the scanning rate, with the oxidation peak shifting to a higher potential and the reduction peak shifting to a lower potential. This behavior is attributed to the strong polarization during the oxidation–reduction process. The results, shown in Figure 5b, were derived by performing a linear fit of the square root of the peak currents for both the oxidation and reduction peaks at each scan rate, as presented in Figure 5a. Both the anode and cathode peak currents were proportional to the scan rate’s square root. Clearly, the oxidation of glucose on the Cu/Fe_3_O_4_/NF(1:1) electrode is a fully diffusion-controlled process [28,29]. It is well known that the amperometric response of a glucose sensor is largely dependent on the applied potential. To determine the optimal potential for catalytic activity, 0.01 mM glucose was continuously added to 0.1 M NaOH solution for amperometric testing, as shown in Figure 5c. The results show that at a potential of 0.55 V, the glucose current response is relatively low. In contrast, applying potentials of 0.6 V and 0.65 V results in a marked increase in the current. However, at 0.65 V, the background current in the response curve is noticeably higher, which interferes with the glucose oxidation signal. Based on these observations, +0.6 V was determined to be the optim26al potential for subsequent experiments. The corresponding calibration curve of the Cu/Fe_3_O_4_/NF(1:1) electrode towards the glucose concentration is shown in Figure 5d. The linear detection range spans from 1 μM to 1 mM, within which the current density exhibits a good linear relationship with glucose concentration. The fitted equation is I (mA/cm^2^) = 1.1891 + 0.01285C (μM), with a correlation coefficient (R^2^) of 0.986. The sensitivity of the non-enzyme Cu/Fe_3_O_4_/NF(1:1) glucose sensor is 12.85 μA·μM^−1^·cm^−2^, and the detection limit is 0.71 μM (3σ/S), where σ represents the standard deviation of the background current, and S is the slope of the calibration curve. We further compared the Cu/Fe_3_O_4_/NF(1:1) electrode with the same composition as reported (Table S3), and it can be seen that our prepared electrode has a bright performance both in the sensitivity and detection limit. These results demonstrate that the Cu/Fe_3_O_4_/NF(1:1) electrode has a low detection limit, making it suitable for effectively detecting glucose concentrations in blood (4–6 mM) and other biological fluids such as saliva and urine.

Additionally, Figure 5e further compares the CV curves of NF, Fe_3_O_4_, Cu, and Cu/Fe_3_O_4_/NF(1:1) electrodes at a glucose concentration of 100 μM. The results show that the NF substrate exhibits no distinct glucose oxidation peak, indicating almost no response to glucose. In contrast, the glucose response current of the Cu/Fe_3_O_4_/NF(1:1) electrode is significantly higher than that of the NF, Fe_3_O_4_, and Cu electrodes, highlighting the superior electrocatalytic performance of the Cu/Fe_3_O_4_ composite material. Generally, the charge transfer resistance was directly correlated with the radius of the Nyquist curve in the AC impedance pattern, where a smaller radius denoted a higher rate of combination. Figure 5f displays the electrochemical impedance profiles of the NF and Cu/Fe_3_O_4_/NF(1:1). The impedance spectra of NF and Cu/Fe_3_O_4_/NF(1:1) were all in a semi-circle, revealing the charge transfer controlled reaction at the electrodes. The radius of Cu/Fe_3_O_4_/NF(1:1) was smaller than that of NF, indicating that its unique multi-dimensional nanostructure resulted in faster charge transport, accelerating glucose adsorption and the transfer of electrons. Based on the above results, several key factors can be attributed to the enhanced electrocatalytic performance of Cu/Fe_3_O_4_/NF(1:1). First, the layered nanosheet structure of Cu/Fe_3_O_4_/NF(1:1) not only increases the exposure of active sites but also facilitates faster electron transfer between the electrolyte and the electrode during the electrocatalytic process. Second, the combination of Cu and Fe_3_O_4_ leverages Cu’s high electrical conductivity while preserving the catalytic activity of Fe_3_O_4_. Finally, the self-supporting structure eliminates the need for adhesives and ensures excellent electrical conductivity between the substrate and the active material.

**Figure 6:**
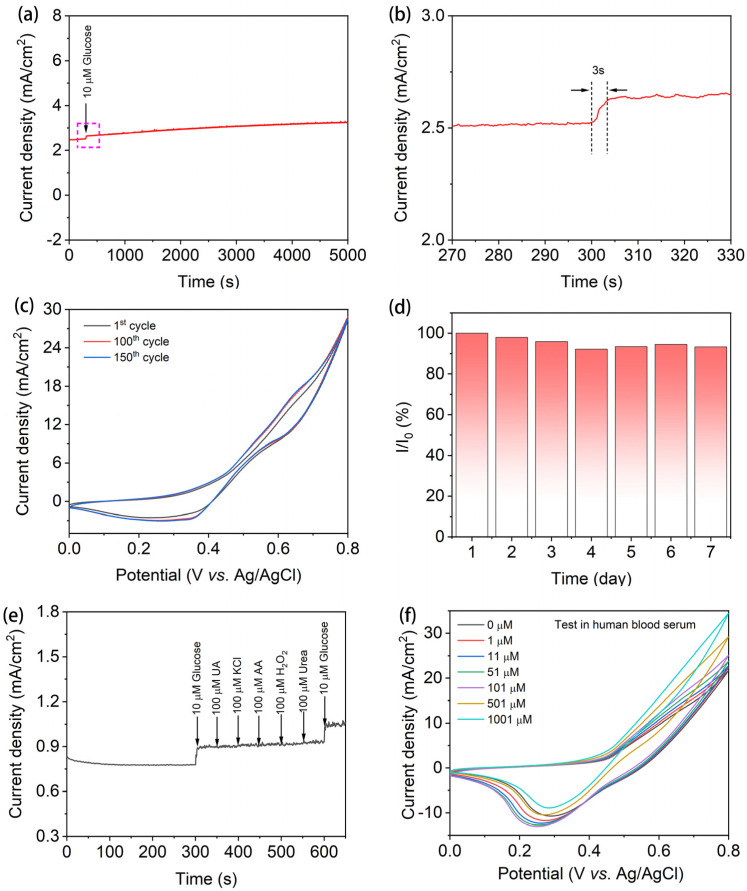
(**a**) The stability test at 5000 s for long runs; (**b**) the response time of the Cu/Fe_3_O_4_/NF(1:1) electrode to achieve a steady-state current; (**c**) CV curves for different cycles; (**d**) long-term stability of the Cu/Fe_3_O_4_/NF(1:1) electrode; (**e**) the anti-interference test of the Cu/Fe_3_O_4_/NF(1:1) electrode in 0.1 M NaOH solution at 0.60 V with the glucose concentration of 0.01 mM and the interferents; (**f**) CV curves of the Cu/Fe_3_O_4_/NF(1:1) electrode in 20 mL of 0.1 M NaOH containing 800 µL of human serum (4.07 mM), with glucose concentrations ranging from 0 to 1001 μM.

The long-term stability and interference ability are important indicators of a sensor. Figure 6a illustrates the I-t curve of the Cu/Fe_3_O_4_/NF(1:1) electrode to 0.01 mM glucose within a 5000 s period. There was a nearly non-existent loss in the current signal over a 5000 s period for 0.01 mM glucose in 0.1 M NaOH at +0.60 V, evidencing exceptional stability of the Cu/Fe_3_O_4_/NF(1:1) electrode. The instantaneous jump of the current signal within 3 s to reach a steady state further demonstrated the high sensitivity of the sensor to glucose detection (Figure 6b). The amperometric response curves of the Cu/Fe_3_O_4_/NF(1:1) electrode after different cycles were investigated, as shown in Figure 6c. The CV curves after 1, 100, and 150 cycles were observed to be nearly constant, with no discernible variation in the current signal. The Cu/Fe_3_O_4_/NF(1:1) composite material was used for glucose detection once a day for 7 consecutive days, totaling seven measurements, to assess the stability of the sensor based on changes in the current response. As shown in Figure 6d, the current response of Cu/Fe_3_O_4_/NF(1:1) fluctuated very little over the 7 days. Even on the 7th day, it maintained 93.3% of its initial current response. This further demonstrates the excellent stability of the Cu/Fe_3_O_4_/NF(1:1) electrode.

As reported, numerous readily oxidative species including ascorbic acid (AA), uric acid (UA), and potassium chloride (KCl) are present in human blood, which may have an impact on the electrochemical response. Since the levels of the interfering agents are significantly lower than those of glucose in a normal physiological state, the anti-interference effect was tested by adding 0.01 mM glucose, 0.1 mM UA, 0.1 mM KCl, 0.1 mM AA, 0.1 mM H_2_O_2_, 0.1 mM urea, and 0.01 mM glucose in that order. Herein, the selectivity of the Cu/Fe_3_O_4_/NF(1:1) was evaluated at a potential of +0.60 V in 0.1 M NaOH solution, as shown in Figure 6e. There was an apparent glucose response, but the interfering species showed no discernible current response. Consequently, the Cu/Fe_3_O_4_/NF(1:1) nanosheet-modified electrode has good selectivity for interfering agents (such as UA and AA), and strong resistance to Cl^−^ poisoning benefits from the complementary effects of Cu and Fe metals as well as excellent electrocatalytic activity toward glucose detection. More importantly, the potential of Cu/Fe_3_O_4_/NF(1:1) for practical applications was also obtained in the real human blood serum, where the glucose concentration change was evaluated by altering the volume of the liquid used for testing. We added 800 µL of human serum (with a blood glucose concentration of 4.07 mM) to 20 mL of 0.1 M KOH electrolyte solution, and subsequently evaluated CV curves of Cu/Fe_3_O_4_/NF(1:1) as the glucose concentration increased in this system. As demonstrated in Figure 6f, the current signals gradually enhanced with the increase in glucose concentration, and its trend of variation is consistent with that in Figure 4a, showing superior sensitivity and accuracy to glucose in human serum. To further assess the practical detection performance of the Cu/Fe_3_O_4_/NF(1:1) electrode, 200, 400, and 800 μL human serum samples (4.07 mM) were added to 20 mL of 0.1 M KOH electrolyte, i.e., human serum was diluted 100-fold, 50-fold, and 25-fold, respectively. And, then, the CV curve was measured (Figure S2). As shown in Table S4, the glucose concentrations in the serum sample measured by the fully automated biochemical analyzer were 4.07 mM, while our sensor readings were 3.52 mM, 4.27 mM, and 3.97 mM according to the standard curve fitting (Figure 5d), with relative standard deviations (RSDs) of 2.02%, 5.15%, and 3.27%, respectively. Therefore, the results obtained with the fabricated sensor show good consistency with the analyzer, demonstrating its potential for glucose detection in real human serum samples.

## 4. Conclusions

In summary, a self-supporting hierarchical Cu/Fe_3_O_4_ nanosheet electrode was fabricated by in situ growth on NF via a hydrothermal method and annealing treatment. The Cu/Fe_3_O_4_/NF sample possesses a large specific surface area, facilitating electron transfer and increasing the number of active sites. In glucose detection applications, the Cu/Fe_3_O_4_/NF electrode has high sensitivity (12.85 μA·μM^−1^·cm^−2^) with a low detection limit of 0.71 μM and fast response speed (~3 s). Furthermore, the Cu/Fe_3_O_4_/NF electrode demonstrates excellent selectivity and stability for glucose, with high anti-interference ability against ascorbic acid, uric acid, urea, KCl, and H_2_O_2_. It also exhibits superior sensitivity and accuracy relative to glucose in human serum. These findings convincingly notarize the potential of the Cu/Fe_3_O_4_/NF composite nanosheets for non-enzymatic electrochemical glucose detection.

## Data Availability

Data are contained within the article.

## Supplementary Materials

The following supporting information can be downloaded at: www.mdpi.com/article/10.3390/nano15040281/s1, Table S1. Atomic ratios of copper species in Cu 2p and Cu LMM. Table S2. The ICP-MS data of Cu/Fe_3_O_4_/NF(1:1). Table S3. Compared performances of Cu/Fe_3_O_4_/NF(1:1) electrodes with other reported Fe-based and Cu-based glucose sensors. Table S4. Glucose determination in human blood serum samples levels (mean ± SD, n = 3). Figure S1. The XPS of Cu LMM spectra over Cu/Fe_3_O_4_/NF(1:1) electrode. Figure S2. CV curves of the Cu/Fe_3_O_4_/NF(1:1) electrode in 20 mL of 0.1 M NaOH containing 200, 400 and 800 µL of human serum (4.07 mM), respectively. Refs. [16,21,23,30,31,32,33,34,35,36,37,38,39,40,41,42] are cited in the Supplementary Materials.
