# Supplementary material for: Self-Supported Cu/Fe3O4 Hierarchical Nanosheets on Ni Foam for High-Efficiency Non-Enzymatic Glucose Sensing
Source: Nanomaterials (Basel). 2025 Feb 12;15(4):281. doi: 10.3390/nano15040281 (PMC11857864; doi:10.3390/nano15040281)
Supplement: Supplementary file 1 [file nanomaterials-15-00281-s001.zip › nanomaterials-3445594-supplementary.pdf]

Supplementary Materials

Self-Supported Cu/Fe_3O_4 Hierarchical Nanosheets on Ni Foam for High-Efficiency Non-enzymatic Glucose Sensing

Jing Xu [1,2], Hairui Cai [1,*], Ke Yu [2], Jie Hou [1], Zhuo Li [3], Xiaoxiao Zeng [1], Huijie He [1], Xiaojing Zhang [1], Di Su [4] and Shengchun Yang [1]

[1] MOE Key Laboratory for Non-Equilibrium Synthesis and Modulation of Condensed Matter, Key Laboratory of Shaanxi for Advanced Materials and Mesoscopic Physics, State Key Laboratory for Mechanical Behavior of Materials, School of Physics, Xi'an Jiaotong University, No. 28 West Xianning Road, Xi'an 710049, China; xujing1@xpu.edu.cn (J.X.); sherlock_hou@stu.xjtu.edu.cn (J.H.); zxxkeep@stu.xjtu.edu.cn (X.Z.); a592003062@stu.xjtu.edu.cn (H.H.); jingfang114love@xjtu.edu.cn (X.Z.); ysch1209@xjtu.edu.cn (S.Y.)

[2] School of Mechanical and Electrical Engineering, Xi'an Polytechnic University, No. 19 Jinhua South Road, Xi'an 710048, China; y19145514@xauat.edu.cn

[3] School of Electrical Engineering, Xi'an Jiaotong University, Xi'an 710049, China; lizhuo3007@stu.xjtu.edu.cn

[4] Shaanxi Hydrogen Energy Research Institute Co., Ltd., Xi'an 712046, China; sudi600@sina.com

* Correspondence: caihairui@xjtu.edu.cn

As shown in Table S1, We carefully examined the atomic ratios in Figure S1 (Cu(I)+Cu(0))/Cu(II) and compared them with the atomic ratios in Figure 1(d). After verification, the two ratios were relatively similar, with values of 1.15 (Cu LMM) and 1.02 (Cu 2p), respectively, indicating a consistency in the distribution of copper oxidation states across the two different analysis methods.

Table S1. Atomic ratios of copper species in Cu 2p and Cu LMM.

Type of XPS spectra	Cu species	Atomic%	Ratio
Cu 2p	Cu^{2+}	50.51	1.02
	Cu^{0}/Cu^{1+}	49.49	
Cu LMM	Cu^{2+}	53.69	1.05
	Cu^{0}/Cu^{1+}	46.31	

Table S2. The ICP-MS data of Cu/Fe_3O_4/NF(1:1).

**Catalyst**	**Fe（ug/mL）**	**Cu（ug/mL）**
**Cu/Fe_3O_4/NF(1:1)**	99.87	162.14

Table S3. Compared performances of Cu/Fe_3O_4/NF(1:1) electrodes with other reported Fe-based and Cu-based glucose sensors.

Electrode materials	Sensitivity(μA μM^{-1} cm^{-2})	Linear range (mM)	Detection (μM)	Ref.
**Cu/Fe_3O_4/NF(1:1)**	**12.85**	**0.001-1**	**0.71**	**This work**
**NiFe(NPs)-PANi**	1.05	0.02-1	0.5	[1]
**CuS@$Ni(OH)_2$(1:2)/CC**	2.171	0.001-0.15	0.19	[2]
**Cu-Co/rGO/PGE**	0.24	0.002-0.08	0.15	[3]
**Fe-doped g-C_3N_4 NPs**	2.4	0.001-1	0.5	[4]
**g-C_3N_4/Fe_2O_3-Cu**	1.067	0.006-2	0.3	[5]
**g-C_3N_4/α-Fe_2O_3**	0.58	0.002-2.4	0.4	[6]
**Cu@Co-MOF/GCE**	2	0.005-1.8	1.6	[7]
**g-C_3N_4-Fe_3O_4**	2.17	0.001-0.14	0.25	[8]
**Fe_3O_4 nanosphere**	6.56	0.1-1.1	33	[9]
**hollow Fe_3O_4 nanospheres**	0.095±0.0054	0-18	19.2	[10]
**Cu_2O-Cu-Au**	1.082	0-4.5	1.71	[11]
**Pt_1/Cu@CuO NWs**	0.852	0.01-5.18	3.6	[12]
**Cu@Ni core–shell nanoparticles**	6.905	0.001-1.63	0.03	[13]
**Cu NPs-LIG**	0.495	0.001-6	0.39	[14]
**FeCo@PCNSs-800**	1.766	0.005-1.7	0.1	[15]

Table S4. Glucose determination in human blood serum samples levels (mean±SD, n=3)

Sample number	Developed glucose sensor (mM)	R.S.D.(%)	Biochemical analyzer (mM)
**1**	3.52±0.071	2.02	4.07
**2**	4.27±0.22	5.15	4.07
**3**	3.97±0.13	3.27	4.07

The Cu LMM spectra were obtained through nonlinear least squares (NLS) fitting using Avantage software. The standard spectra for Cu, Cu_2O, and CuO were sourced from the Avantage software database, with their shapes consistent with those reported in the literature[16].

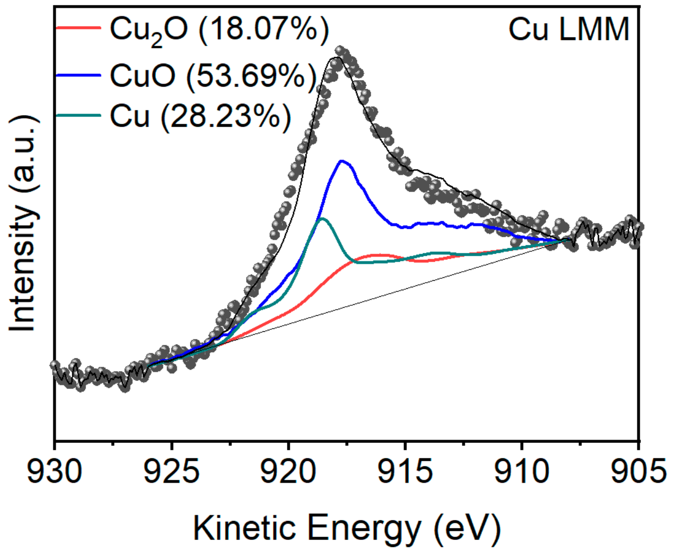

Figure S1. The XPS of Cu LMM spectra over $Cu/Fe_3O_4/NF(1:1)$ electrode.

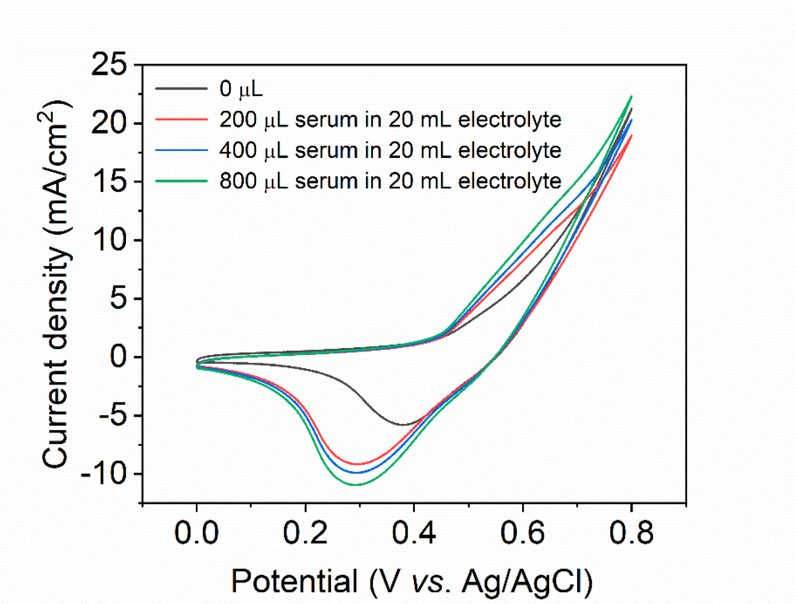

Figure S2. CV curves of the Cu/Fe_3O_4/NF(1:1) electrode in 20 mL of 0.1 M NaOH containing 200, 400 and 800 μL of human serum (4.07 mM), respectively.

Reference

[1] D. Lakhdari, A. Guittoum, N. Benbrahim, O. Belgherbi, M. Berkani, Y. Vasseghian, N. Lakhdari, A novel non-enzymatic glucose sensor based on NiFe(NPs)–polyaniline hybrid materials, Food and Chemical Toxicology, 151 (2021) 112099.

[2] G. Siva, M.A. Aziz, G. Gnana kumar, Engineered Tubular Nanocomposite Electrocatalysts Based on CuS for High-Performance, Durable Glucose Fuel Cells and Their Stack, ACS Sustainable Chemistry & Engineering, 6 (2018) 5929-5939.

[3] K. Justice Babu, S. Sheet, Y.S. Lee, G. Gnana kumar, Three-Dimensional Dendrite Cu–Co/Reduced Graphene Oxide Architectures on a Disposable Pencil Graphite Electrode as an Electrochemical Sensor for Nonenzymatic Glucose Detection, ACS Sustainable Chemistry & Engineering, 6 (2018) 1909-1918.

[4] Y. Peng, X. Yu, W. Yin, W. Dong, J. Peng, T. Wang, Colorimetric Assay Using Mesoporous Fe-Doped Graphitic Carbon Nitride as a Peroxidase Mimetic for the Determination of Hydrogen Peroxide and Glucose, ACS Applied Bio Materials, 3 (2020) 59-67.

[5] L. Liu, M. Wang, C. Wang, In-situ synthesis of graphitic carbon nitride/iron oxide−copper composites and their application in the electrochemical detection of glucose, Electrochimica Acta, 265 (2018) 275-283.

[6] L. Liu, J. Wang, C. Wang, G. Wang, Facile synthesis of graphitic carbon nitride/nanostructured α-Fe2O3 composites and their excellent electrochemical performance for supercapacitor and enzyme-free glucose detection applications, Applied Surface Science, 390 (2016) 303-310.

[7] Z.-Z. Ma, Y.-S. Wang, B. Liu, H. Jiao, L. Xu, A Non–Enzymatic Electrochemical Sensor of Cu@Co–MOF Composite for Glucose Detection with High Sensitivity and Selectivity, Chemosensors, 2022.

[8] K. Tian, H. Liu, Y. Dong, X. Chu, S. Wang, Amperometric detection of glucose based on immobilizing glucose oxidase on g-C3N4 nanosheets, Colloids and Surfaces A: Physicochemical and Engineering Aspects, 581 (2019) 123808.

[9] J. Xu, Y. Sun, J. Zhang, Solvothermal synthesis of Fe3O4 nanospheres for high-performance electrochemical non-enzymatic glucose sensor, Scientific Reports, 10 (2020) 16026.

[10] F. Zhou, J. Wang, Y. Tang, X. Song, W. Zhou, Y. Li, F. Gao, Enhanced sensing performance of flexible non-enzymatic electrochemical glucose sensors using hollow Fe3O4 nanospheres of controllable morphologies, Ceramics International, 50 (2024) 38009-38021.

[11] F. Pu, H. Miao, W. Lu, X. Zhang, Z. Yang, C. Kong, High-performance non-enzymatic glucose sensor based on flower-like Cu2O-Cu-Au ternary nanocomposites, Applied Surface Science, 581 (2022) 152389.

[12] Y. Zhao, Y. Jiang, Y. Mo, Y. Zhai, J. Liu, A.C. Strzelecki, X. Guo, C. Shan, Boosting Electrochemical Catalysis and Nonenzymatic Sensing Toward Glucose by Single-Atom Pt Supported on Cu@CuO Core–Shell Nanowires, Small, 19 (2023) 2207240.

[13] A. Farid, A.S. Khan, M. Javid, M. Usman, I.A. Khan, A.u. Ahmad, Z. Fan, A.A. Khan, L. Pan, Construction of a binder-free non-enzymatic glucose sensor based on Cu@Ni core–shell nanoparticles anchored on 3D chiral carbon nanocoils-nickel foam hierarchical scaffold, Journal of Colloid and Interface Science, 624 (2022) 320-337.

[14] Y. Zhang, N. Li, Y. Xiang, D. Wang, P. Zhang, Y. Wang, S. Lu, R. Xu, J. Zhao, A flexible non-enzymatic glucose sensor based on copper nanoparticles anchored on laser-induced graphene, Carbon, 156 (2020) 506-513.

[15] M. Li, P. Dong, Y. Zhang, Facile design and synthesis of ultrafine FeCo nanocrystallines coupled

with porous carbon nanosheets as high efficiency non-enzymatic glucose sensor, Journal of Alloys and Compounds, 810 (2019).
[16] M.C. Biesinger, Advanced analysis of copper X-ray photoelectron spectra, Surface and Interface Analysis, 49 (2017) 1325-1334.
